# Impact of the Pandemic on the Teaching and Research Staff at a Technological University in Spain: Deepening the Gender Gap

**DOI:** 10.3390/ijerph19116417

**Published:** 2022-05-25

**Authors:** Marta Peña, Noelia Olmedo-Torre, Olga Alcaraz, Juan A. Chavez-Dominguez, José López, Luis Eduardo Mujica

**Affiliations:** 1 Department of Mathematics, Universitat Politècnica de Catalunya, 08034 Barcelona, Spain; marta.penya@upc.edu (M.P.); luis.eduardo.mujica@upc.edu (L.E.M.); 2 Department of Graphic and Design Engineering, Universitat Politècnica de Catalunya, 08034 Barcelona, Spain; 3 Department of Physics, Universitat Politècnica de Catalunya, 08034 Barcelona, Spain; olga.alcaraz@upc.edu (O.A.); josep.lopez-lopez@upc.edu (J.L.); 4 Electronic Engineering Department, Universitat Politècnica de Catalunya, 08034 Barcelona, Spain; juan.antonio.chavez@upc.edu

**Keywords:** gender, higher education, pandemic, professional and academic labour, social vulnerability, teaching and research staff

## Abstract

The alteration of the educational model caused by the COVID-19 pandemic has not affected all university faculty equally. This work explores the academic, digital and gender inequalities caused by the pandemic on the teaching and research staff of a technological university for STEM (Science, Technology, Engineering and Mathematics) disciplines in Spain, the Universitat Politècnica de Catalunya—BarcelonaTech (UPC). The study considers an anonymous survey with a non-probabilistic voluntary sample (*n* = 355). The results of the survey reveal that, over these months, the teaching and research staff of the university, regardless of gender, has significantly increased its academic activity due especially to the number of hours devoted to virtual teaching compared to its teaching dedication in a situation of normalcy. This study shows that the lockdown has strongly affected women who are more vulnerable to crisis. In particular, the negative impact on research has been higher in female faculty staff from the UPC, who already face disparities regarding promotion and, during lockdown, stated more difficulties with household work reconciliation. From the results of this study, it is possible to conclude that the COVID-19 pandemic has deepened the gender gap in the academic field.

## 1. Introduction

### 1.1. Theoretical Framework

The state of alarm and subsequent lockdown decreed by most governments due to the arrival and spread of COVID-19 has caused an unprecedented situation in the world that has had and will have very important repercussions for the whole of society. In particular, the temporary cessation of face-to-face activity throughout the educational community has suddenly caused an alteration in the teaching model that has affected teachers, students, and administration and service personnel of higher education institutions. The impact of this alteration has been highly variable in the faculty staff across different higher education institutions. This impact depends largely on the university’s ability to keep its academic activity alive, the ability to react and rethink the teaching model during the lockdown, the technological capacity available in that moment and the financial solvency of the higher education institution to face investments in new equipment and hiring of personnel [1,2,3,4].

Suddenly, face-to-face universities around the world have been forced to progressively adapt new learning activities and different evaluation methodologies [5,6] derived from distance education [7,8,9,10] which, in principle, has meant the use of human resources. Furthermore, the impact of this precipitate transition to online education methods in engineering studies is still not fully known [11], and many challenges related to online education methods are still to be addressed [12]. 

Referring to medical schools, Burki [13] states that online learning is not a substitute for laboratory work. There are also examples where instructors have struggled to deal with COVID-19 restrictions in subjects that traditionally require face-to-face labs, such as the case of [14] chemistry experiments. Likewise, there are some situations such as the previous use of B-learning methodologies that seem to have worked as a facilitator of the response and sudden transition to online study due to COVID-19, as pointed out by [15] at a Spanish university. Practical applications and teamwork are a fundamental part to achieve the competency objectives of the study plans, especially in STEM (Science, Technology, Engineering and Mathematics) fields, as Souza et al. [16] remarks. Achieving a transition to online instruction in a short space of time poses a series of challenges for teachers as the workload impacts on a personal and social level with consequences that are still unpredictable; some resources and recommendations specific in physiology are provided by Petzold [17]. Added to this are the containment and mitigation strategies adopted by national governments and the measures taken by higher education authorities that have radically altered the nature of teaching and academic work, with social distancing, travel restrictions, isolation and quarantine procedures, campus closures and border closures being some of these measures.

The consequences of these abrupt changes have been felt by higher education teachers who have suddenly seen how the change in the teaching model has meant adapting their academic work to the institutional responses to the crisis. For example, Dwivedi et al. [18] indicate, referring to information management research, that this situation affects the increase in workload; Wang et al. [19] highlight the difficulties in the reconciliation of professional activity with personal life, especially taking care of children; and Brooks et al. [20] point out a very significant stress situation during the quarantine.

Lockdown, as an unpleasant experience, can involve boredom and uncertainty [20,21] and adverse psychological effects in people who suffer from it [22]. Isolation poses a significant challenge to the teaching experience [23]. In situations of lockdown, contact and exchange of experiences with other teachers can reduce the negative impact of lockdown on the mental health of the group [24,25,26]. On the contrary, the lack of these relationships with the academic community is related to greater academic stress [26,27,28], so the adverse psychological effects of lockdown may increase if there is a lack of interaction with fellow teachers. 

Hockberger [29] and Dubosh [30] state that life in academics is working to adapt and adapting to improve. So teachers are willing to use both new and existing resources and methodologies to achieve their teaching goals and engage students through online teaching [31,32]. In addition, faculty members are actively engaged in updating their knowledge on distance education [33], keeping mental activeness among students [34], and writing research papers. Rapid (and often improvised) adjustments to work methodology, with uncertain measured results and often based on intuition, have caused stress and nervousness in teachers. It has taken a lot of effort to maintain (or even improve) academic achievement and student satisfaction [35,36]. The workload and the stress endured has been much greater than in the face-to-face classes, as Wilson et al. [32] report.

The lockdown has caused teachers to be more attentive to the factors that cause variations in the teaching-learning results due to the adequacy of the resources available to the students, the suitability of the active learning methodologies used in each subject and the technological differences between academic fields [37,38,39]. In addition, there is the fact that much of the academic staff required levels of technological competence higher than those they had previously acquired and that some degrees were more difficult to adapt to online education [40]. Many teachers who converted their classes to distance often did not have the necessary expertise for online teaching and learning pedagogy and found this task very intimidating [41]. This was complicated by the fact that the majority of higher education teachers had never taken a course in teaching, much less on instructional design for online learning [42].

### 1.2. Gender Inequalities

“Women rival men in scientific research publications and citations” was published by Nature Index on 17 March 2020 [43]. On May 19, Nature denounced the opposite trend: “The decline in women’s research production during the coronavirus pandemic” [44].

During the lockdown, women’s responsibilities increased as they desperately tried to balance teaching and working from home with increased caregiving responsibilities. This included full-day childcare due to school and daycare closures, homeschooling, and the cooking and cleaning associated with having the family home all day, every day. They take on more service work than men and are less protective of their research time [44]. It is not that men do not help with all of these tasks, or that they are not individually overwhelmed by work and personal life as well. However, women were already juggling more domestic and affective, or emotional, work with their real work before the pandemic [43].

The pandemic exacerbated gender imbalances in scientific research. The loss of time in research due to the accumulation of housework has precipitated numerous findings [45,46,47,48] showing that women are underrepresented as authors of research articles in many scientific areas, particularly in senior authorship positions. During the pandemic they represented around a third of all authors who published articles related to COVID-19. The representation of women is even lower for the first and last positions of authorship. Megan Frederickson, University of Toronto, Canada, looked at preprint servers to see if women were publishing fewer studies than before lockdown, and confirms, in all disciplines, the publication rate of women has decreased in relation to that of men in the midst of the pandemic [49].

The low percentage of female authors is consistent with similar studies in other research areas. As Andersen et al. [50] state, in an analysis of 20 years of publication in high-impact medical journals, female first authors were observed in 34% of the articles and this has been decreasing in that period. Female first authors in infectious disease publishing topics decreased by 4% from 1994 to 2014. Current restrictions imposed during the COVID-19 pandemic may have further contributed to this decline. The same study estimates that the proportion of articles on COVID-19 with a woman as the first author was 19% lower than that of articles published in the same journals in 2019.

On the other hand, women researchers in economic fields had a drop in the production of preprints and reports registered in March and April 2020. Similarly, in those months, male authors in arXiv and bioRxiv increased at a higher rate than female authors, as Vincent-Lamarre et al. [51] report. Amano-Patino et al. [52] suggest that the research productivity of women economists has been disproportionately affected by lockdown measures. It is mostly senior male economists who are carrying out research on COVID-19.

Although the pandemic has affected all of society equally, the crisis has not affected both sexes equally. Historically, crises affect gender equality negatively. Men and women have suffered from the pandemic differently, even in countries with well-prepared infrastructures to deal with emergencies of this type, as Rubery et al. [53] remark.

With the arrival of COVID-19, in a short time, women have faced a short-term reorganization of attention to daily household tasks and time spent at work, as Cesaroni et al. [54] manifest. Scientific literature has found in research that women take on most of the household chores and childcare, thus seeing a 30% reduction in paid work time and, especially mothers, experiencing a further dramatic impact on their well-being during the pandemic, especially when the usual support networks, such as grandparents, friends and neighbours, can no longer help with childcare [55]. Women must face the balance between work and personal life because gender issues before the confinement were not ideal with respect to women’s rights and gender equality was one of the most urgent societal challenges before the pandemic, as Cesaroni et al. [54] report. Evidence from various studies [46,56,57] suggests that female academics with homecare responsibilities are being disproportionately impacted during the pandemic. Other universities are also reported to have shirked their responsibility to ensure the full participation of women in the workforce [58].

Many investigations reported that in recent months, women are submitting and publishing fewer manuscripts for publication than men. In the long term, this change in productivity will affect their careers, as Minello [46] and Oleschuk [59] state. This trend is reflected in the number of publications that are uploaded to prepress servers for STEM disciplines. According to Frederickson [60], male author submissions between March and April 2020 increased 6.4% on arXiv, while female submissions increased just 2.7% compared to the same period last year. Squazonni et al. [61] calculated the change in submissions during the initial quarantine period using the individual as the unit of analysis. They found that, while article submissions increased for all, they did so at a significantly higher rate among men as reported by other, similar research. Moreover, Krukowski et al. [62] and Matulevicius et al. [63] found that there were differences by gender in academic productivity in STEM and medicine faculties, and Amano-Pariño et al. [64] show that female economists were engaging in less research during the pandemic. Collins et al. [65] found that women, and particularly mothers, had reduced their working hours more than fathers. These findings of a “maternity penalty” are not new and suggest that women’s contribution to the household and childcare tasks have increased with the pandemic, particularly with regard to the responsibility of homeschooling [66,67]. This shows that a greater role of domestic work is a factor that contributes to lower rates of academic publication during the pandemic. All of these data suggest that women and men have not spent their time in the same way during the pandemic.

This crisis affects the position and future opportunities of women in all STEM fields, among other discilplines. Despite continued slow progress in increasing the representation of women in academia, women remain significantly underrepresented at higher levels, as Pardhan [68] states, particularly in sciences and engineering, as Caroline et al. [69] shows from a study at a chemical department of a Swedish university. The higher productivity of men’s scientific publications in STEM fields is mainly due to their greater representativeness compared to women, who are significantly underrepresented in STEM fields globally, as is reported in [70].

### 1.3. Context of the Study

Universitat Politècnica de Catalunya—BarcelonaTech (UPC) is a Spanish public university specialised in the fields of engineering, architecture and ICT (information and communication technology). In 2020, it had 28,000 undergraduate and master’s degree students enrolled (28% women and 72% men).

**Figure 1:**
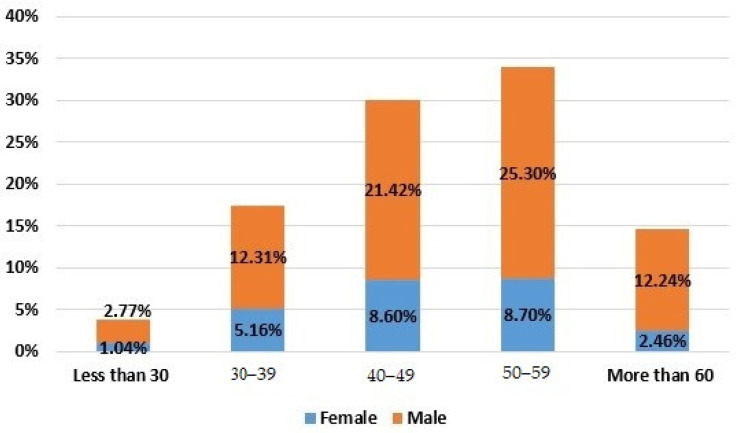
Distribution of the teaching and research staff of the Universitat Politècnica de Catalunya by age and gender.

**Figure 2:**
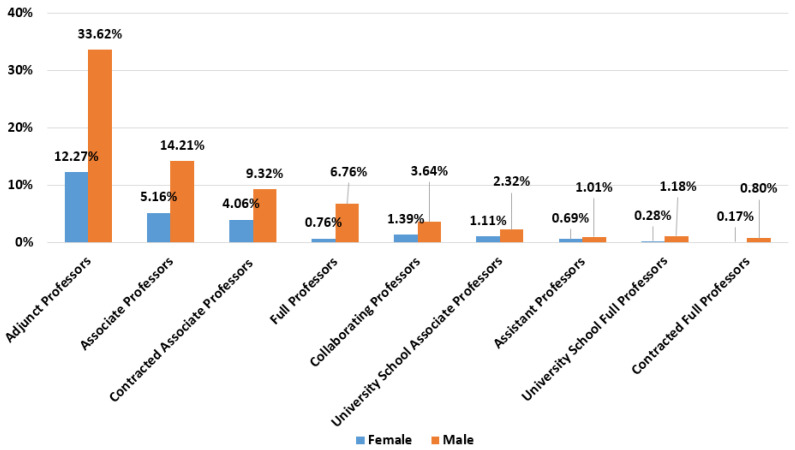
Distribution of the teaching and research staff of the Universitat Politècnica de Catalunya by job category and gender.

Regarding the teaching and research staff of UPC, its distribution by age and gender is shown in Figure 1. A description of the different job categories of the teaching and research staff is shown in Table 1. Figure 2 shows the distribution of the teaching and research staff by job category and gender.

### 1.4. Study Objectives and Research Questions

This work considers the short-term effects of the pandemic with a focus on the impact of gender and other inequalities among the faculty. It focuses on these research questions:

- How does the virtualization of teaching and research due to the pandemic affect work habits?
- What is the degree of satisfaction with academic activities?
- How do faculty staff reconcile personal life and professional activity?
- Are there differences between female and male teaching and research staff in the pre- vious questions?

To understand the difficulties and changes in work habits that virtualization of teaching and research has meant, a survey was launched to the faculty from the Universitat Politècnica de Catalunya. To empirically answer the above research questions and in order to provide insights in the impact of the pandemic on faculty, this study presents several analyses. Firstly, a quantitative analysis is collected in a cross-sectional survey of a group with a non-probabilistic voluntary sample. Secondly, the analysis is also disaggregated by gender, in order to detect significantly different answers from female and male faculty staff. Thirdly, a qualitative analysis of the open-field questions of the previous survey is presented.

## 2. Methodology

A voluntary and anonymous online survey of a cross-sectional and analytical-descriptive type, mostly with closed questions, was sent by email in June 2020. The Google Forms^®^ form has been used for the survey among teaching and research staff who were working at UPC the second quarter of the 2019/2020 academic year. The objective of this survey was to know the opinions of the faculty staff about how they faced the changes in work habits that virtualization of teaching and research has meant. The survey was sent to a total of 2885 teachers, where 26% were women and 74% men. With the data obtained, a quantitative study was carried out analysing the closed questions and a qualitative analysis for the open field questions. Moreover, the results were analysed disaggregated by gender.

The survey asked general questions (Gender, Age, Job category), aspects related to the workload of virtual teaching (Time spent on virtual teaching compared to face-to-face teaching, Increase in student inquiries by email, Weekly hours devoted to student consultations by videoconference, Increase in student consultations for Bachelor/Master Thesis), reconciliation of virtual teaching with personal life, and satisfaction of the teaching task (Teacher work satisfaction, Percentage of subject covered, Student learning satisfaction) impact on research and work equipment. Moreover, the survey included an optional open field to add comments. Table 2 shows the most important design aspects of the survey and Table 3 shows a global description of the questions posed.

For further statistical analysis, answers must be converted to numerical values. Questions Q1.a, Q1.b, Q1.c and Q4 are not ordinal variables, so they are not used. Questions Q8, Q10, Q11 contain numerical values that do not need any conversion to be analyzed; however, questions Q2, Q3, Q5, Q6, Q7 and Q9 include qualitative values that can be converted to numerical ones since the answers have an order that can be easily quantified. For this, a conversion to numerical values for each of the answers has been applied based on the following assignments:

- Q2 (Less than face-to-face ↔ 1, The same ↔ 1, 25% more ↔ 2, 50% more ↔ 3, 75% more ↔ 4, 100% or even more ↔ 5)
- Q3 (Without any other difficulty ↔ 1, With some more difficulties ↔ 2, With many more difficulties ↔ 3)
- Q5 (They have not increased ↔ 1, The increase was less than 50% ↔ 2, The increase was between 50% and 100% ↔ 3, The increase was over 100% ↔ 4)
- Q6 (Zero ↔ 1, From 0 to 3 h per week ↔ 2, From 3 to 6 h per week ↔ 3, More than 6 h per week ↔ 4)
- Q7 (It has not increased ↔ 1, It has doubled ↔ 2, It has tripled ↔ 3)
- Q9 (Less than 80% ↔ 1, 80% ↔ 2, 90% ↔ 3, 100% ↔ 4, 110% or even more ↔ 5)

### 2.1. Internal Consistency Reliability

In order to make sure that all of the items really do reflect the same thing, meaning that responses to different items do not contradict one other, a test of internal consistency reliability is performed. Considering the nature of the data (unidimensional ordinal data and not ceiling effects), and according to Viladrich et al. [71], to estimate the internal consistency reliability, we chose the essentially tau-equivalent measures as the most suitable model. In coherence with the fitted measurement model, the proper estimator was the nonlinear structural equation modelling reliability coefficient developed by Green and Yang $-\omega \left(\rho_{NL}\right)$ [72].

Considering all variables, the value of $\omega \left(\rho_{NL}\right)$ is 0.6649823. If those variables concerning to workload are removed (Q2, Q5, Q6 and Q7), this means that the reliability is studied for items related to Reconciliation, Satisfaction and Research, and the index increases to 0.7333005. These values are within accepted standards in the scale development process, confirming in this way that responses are not contradictory.

### 2.2. Qualitative Analysis

A qualitative analysis using the constant comparison technique was conducted to analyze the replies of the open field question. It was necessary to codify clearly each different reason given in the responses and to identify when a response refers to each reason. An abductive methodology was used to define these codes; that is, codes emerged from the data iteratively. Firstly, half of the dataset was read to enable a list of codes to be identified. Then, by using these previously identified codes, the entire dataset was processed. When all the answers had been read, new reasons were found that had to be coded during the analysis.

## 3. Results

### 3.1. General Results

#### 3.1.1. Initial Aspects

There was a total of 355 responses (12.3% of the total teaching and research staff), 122 were women (34.4%), 232 men (65.5%) and 1 did not answer the question (0.002%).

**Figure 3:**
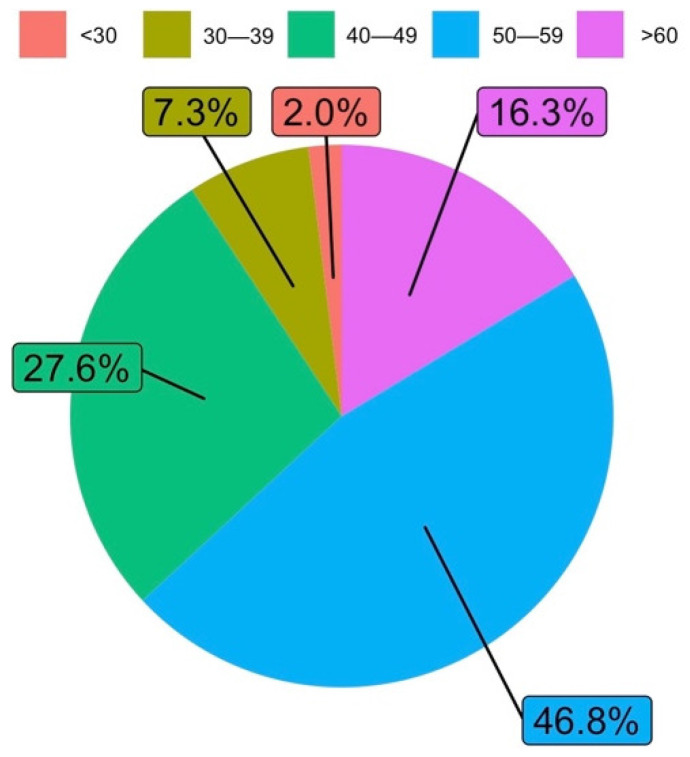
Age groups (answers to Q1.b).

The largest age group that responded to the survey was between 50 and 59 years old (46.8%), followed by those aged 40 to 49 years old (27.6%), and then those over 60 years old (16.3%). Figure 3 shows in detail the answers according to the different age groups.

**Figure 4:**
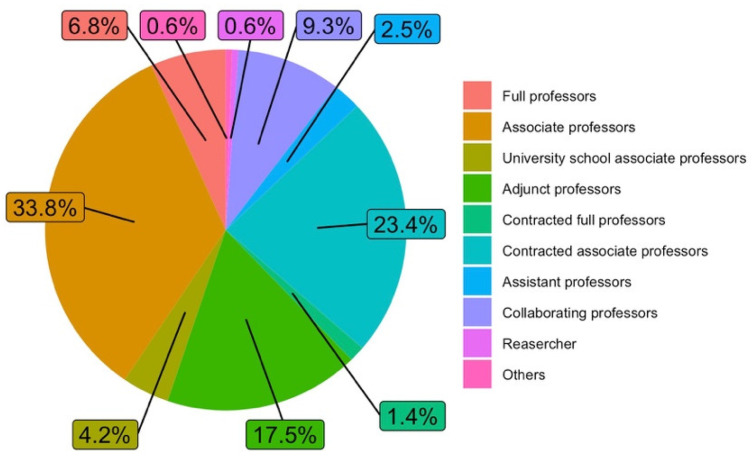
Job categories (answers to Q1.c).

By job category, the largest group that answered the survey was the Associate Professor (statutory staff), with 33.8% of the answers obtained, followed by the Associate Professor (contractual staff), with 23.4%, and then the Assistant Professor, with 17.5%. Figure 4 shows the participation of the different job categories in the survey.

#### 3.1.2. Workload

**Figure 5:**
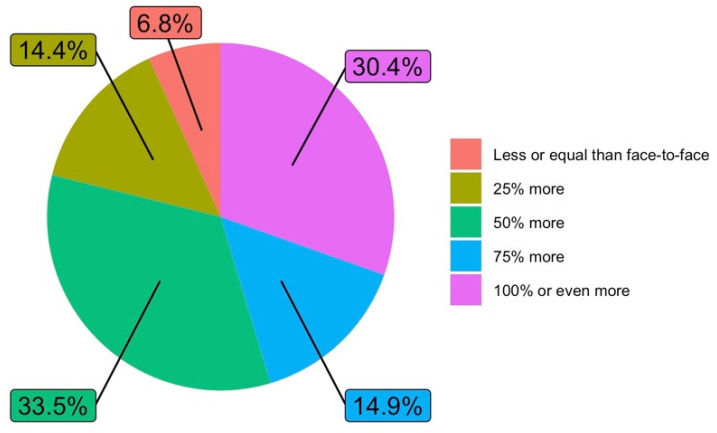
Workload of virtual teaching compared to face-to-face teaching (answers to Q2).

In relation to the volume of work devoted to virtual teaching compared to the volume of work devoted to face-to-face teaching in a normal situation (Q2), 30.4% of the answers stated that the increase in time spent had been 100% or even more. The sum of those who claimed to have spent between 50% and 100% or more is 78.8%. Figure 5 details the total responses obtained on the workload.

The number of hours spent by teachers on student inquiries by email and/or videoconference (Q5, Q6) and the frequency of inquiries about Bachelor or Master Theses (Q7) were two of the most worrying aspects. Regarding the increase in e-mail inquiries in this non-face-to-face teaching period, the sum of teachers who stated that they had increased between 50% and 100% or even more was 49.6%; 52.1% stated that the hours devoted to student consultations by videoconference had been between 0 and 3 h per week. The questions related to the Bachelor or Master Theses have had a diversity of answers with different casuistry, of which it stands out that for 31.2% of the answers, the teaching staff considers that the frequency of the consultations had not increased, and a 23.9% answered that the frequency of consultations had doubled.

#### 3.1.3. Reconciliation

**Figure 6:**
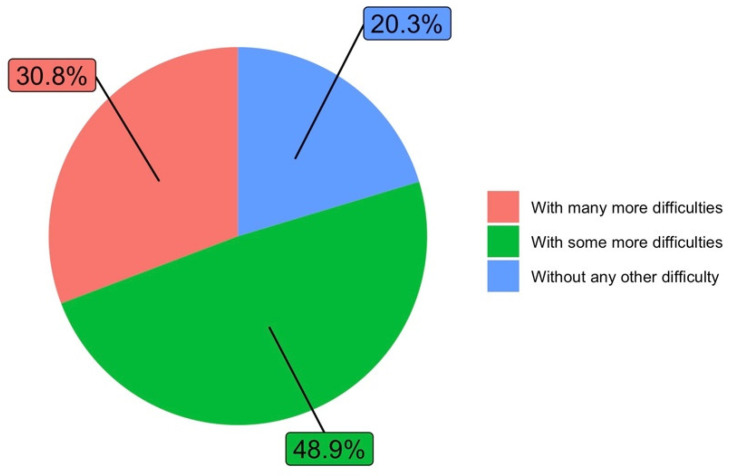
Reconciliation of virtual teaching activity with personal life compared to reconciliation in standard conditions (answers to Q3).

Regarding the reconciliation of teaching activity during the health crisis with personal life, faculty were asked “How do you reconcile teaching activity throughout the health crisis with personal life with respect to the normal situation?” (Q3). Almost half of the respondents stated that reconciliation had occurred with some more difficulties with respect to the normal situation. Only 20.3% said they had no difficulty with reconciliation. Figure 6 shows the answers to this question.

#### 3.1.4. Resources

Regarding the equipment needed to develop online teaching (Q4), the main shortcomings detected by teachers have been the lack of a space, room or office, where they can perform their tasks (42%) and also a suitable table and chair (50%). In reference to the technological elements, the main shortcomings have been the computer (33%), microphone and/or speakers (31%), a good network connection (27%), the webcam (25%) and the software (21%). Others that are also indicated with lower incidence are: the digitising tablet (9%), the blackboard (4%), keyboard and/or large monitor (4%), the lighting of the workspace (2%) and the printer and/or scanner (1%).

#### 3.1.5. Satisfaction

**Figure 7:**
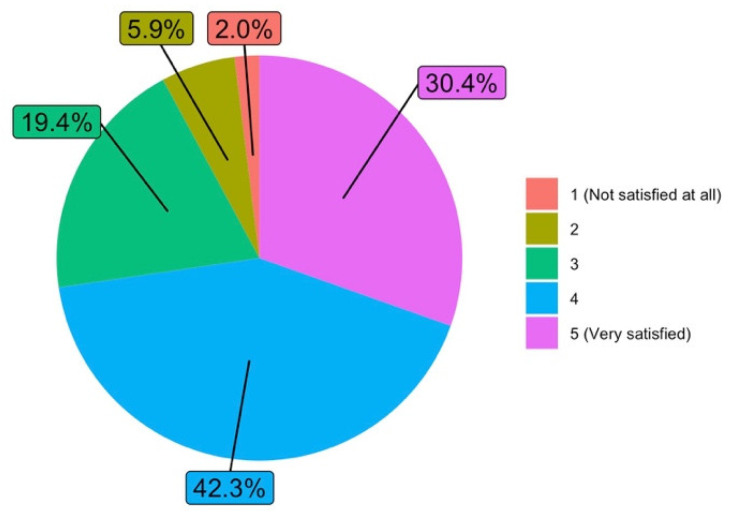
Degree of satisfaction with the work carried out as a teacher during the lockdown period (answers to Q8).

The answers obtained are particularly relevant in relation to the degree of satisfaction with the work carried out as a teacher during the lockdown period (Q8), where 72.7% were “Satisfied” or “Very satisfied”. Figure 7 shows the answers to this question.

**Figure 8:**
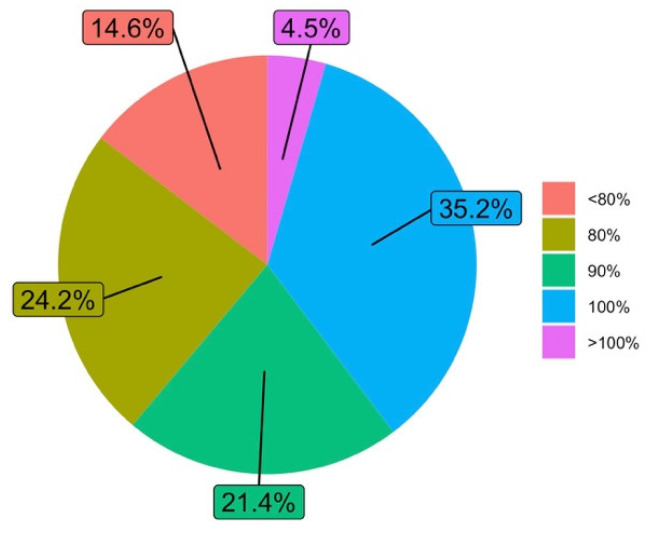
Percentage of the subject that faculty managed to teach with the same depth with respect to previous courses (answers to Q9).

With respect to the percentage of a subject that faculty managed to teach in comparison with previous courses (Q9), it is found that one third of the answers indicate that they have been able to meet the syllabus of the subjects, but the other two thirds state that they have fallen below 100% of the achievement of the syllabus, which may be related to the difficulties in developing the subjects in a virtual environment. However, almost two-thirds of the responses show that they have reached 90% of the syllabus or higher, which must be assessed very positively. See Figure 8.

**Figure 9:**
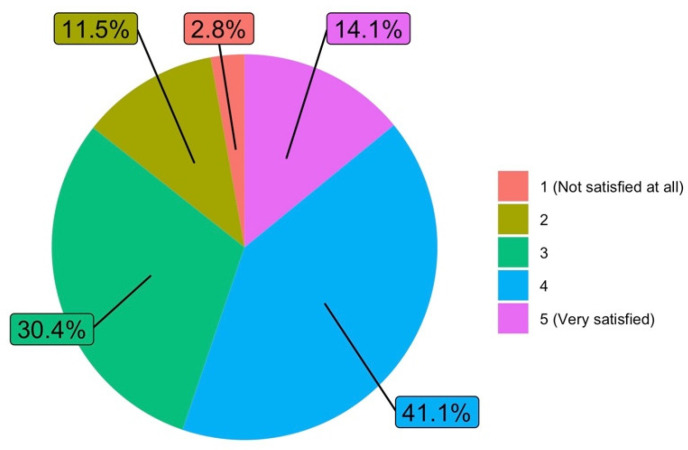
Degree of satisfaction of the teaching staff with the learning achieved by students (answers to Q10).

Regarding the degree of satisfaction of the teaching staff with the learning achieved by students (Q10), the highest scores were “Satisfied” and “Neither satisfied nor dissatisfied”, which were in the order of 70%. A comment should be made on why there were so few (14.1%) “Very satisfied” answers. The situation of lockdown has meant that the assessment has not taken place in appropriate conditions; evaluation criteria have changed and the conditions for carrying out many tests mean that the results obtained from the evaluations, although positive, leave some doubts. See Figure 9.

#### 3.1.6. Research

**Figure 10:**
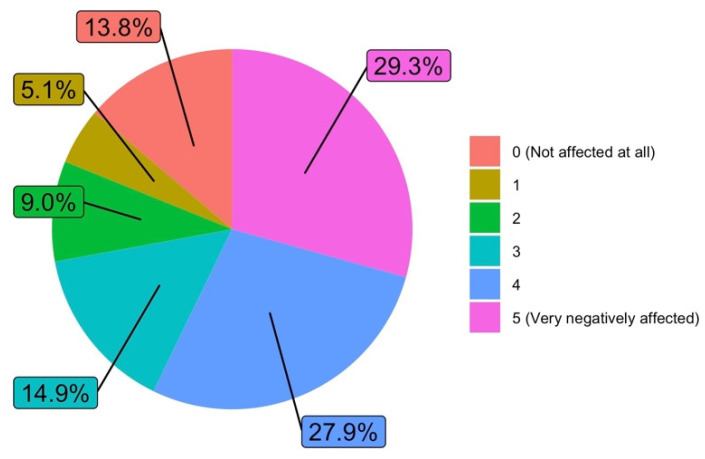
Effects on research due to virtual teaching dedication (answers to Q11).

In relation to the impact on research due to virtual teaching (Q11), only 13.8% of teachers stated that their research was “Not affected”. More than half (57.2%) of teachers said that their research had been “Negatively” or “Very negatively affected”. Figure 10 details the responses related to the impact on research.

### 3.2. Analysis of Results Disaggregated by Gender

The following results are the results of the survey disaggregated by gender. Only those questions in which significantly different responses have been obtained for each gender are shown.

#### 3.2.1. Initial Aspects

**Figure 11:**
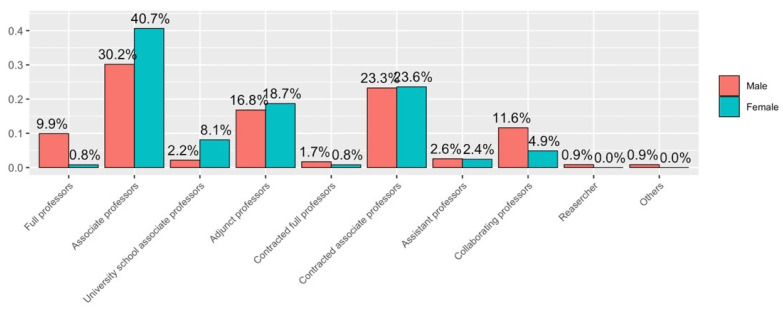
Answers to Q1.c disaggregated by gender.

By job category, in this distribution (Figure 11) the glass ceiling can be recognised, since in both genders the sum of the percentages of the categories Full Professor, Contracted Full Professor, Associate Professor and Contracted Associate Professor is almost identical, but in the case of the male gender it is possible to see the difference in ratios, with male numbers of Full Professors and Contracted Full Professors of 23 (9.9%) and 4 (1.7%) respectively, while in the female gender, it is a very small sample: 1 (0.8%) and 1 (0.8%).

#### 3.2.2. Workload

**Figure 12:**
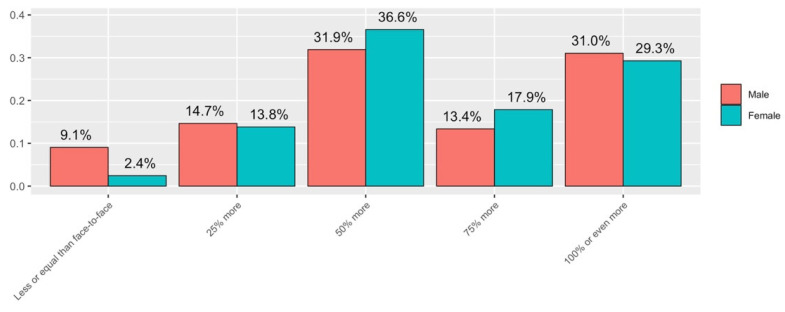
Answers to Q2 disaggregated by gender.

In relation to the volume of work devoted to virtual teaching compared to the volume of work devoted to face-to-face teaching in a normal situation (Q2), as can be seen in Figure 12, more than three-quarters of those surveyed considered that the volume of time spent on virtual teaching compared to the normal situation has increased by at least 50%. It can be seen that women have spent 50% or more time in virtual teaching (85% of women) compared to face-to-face teaching than men (75% of men).

**Figure 13:**
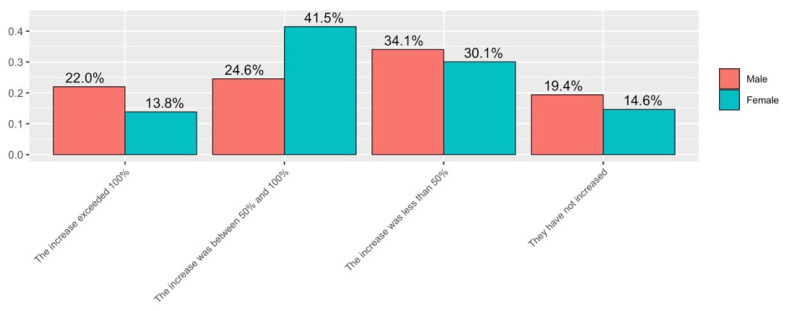
Answers to Q5 disaggregated by gender.

Related to the number of hours spent by teachers on student inquiries by email in the period of virtual teaching (Q5), there was some difference in the answers for both groups (see Figure 13). In the case of women, almost two thirds reported that the number of consultations has increased by between 50% and 100% (41%) or even more than 100% (13.9%). The remaining of responses are distributed among those who state that not increased the number of queries have not increased or that they have increased slightly (below 50%). In the case of men, the most common answer is that the increase has been below 50% (34.1% of responses). The other answers have been distributed evenly. More than half of the responses state that queries have either not increased, or that the increase has been below 50%.

#### 3.2.3. Reconciliation

**Figure 14:**
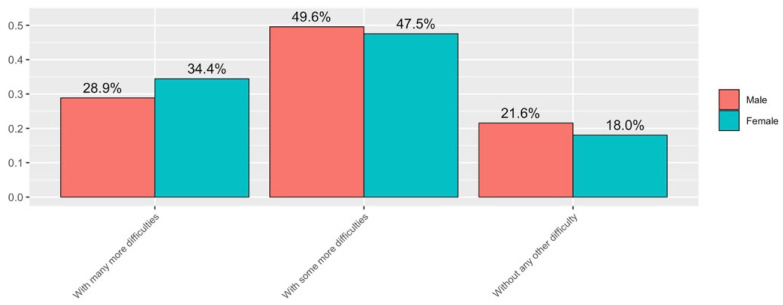
Answers to Q3 disaggregated by gender.

Regarding the reconciliation of teaching activity during the health crisis with personal life (Q3), the answers to this question were very similar and almost independent of gender (see Figure 14). However, women’s responses were more likely to identify that they had more difficulties in reconciling teaching activity with personal life throughout the health crisis compared to men’s responses. Overall, it is found that, regardless of gender, more than 80% of those who completed the survey have reconciled with many or some other difficulties with respect to the normal situation.

#### 3.2.4. Satisfaction

**Figure 15:**
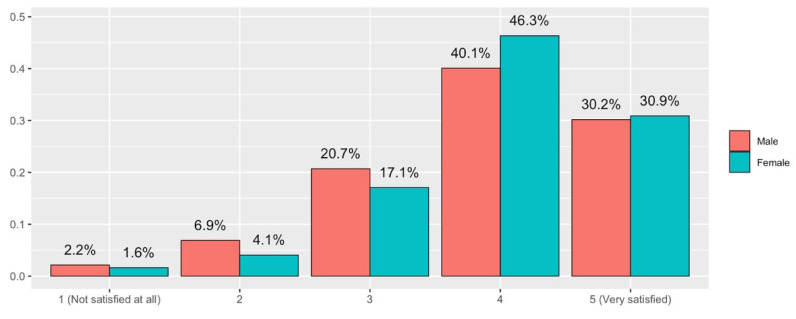
Answers to Q8 disaggregated by gender.

In relation to the degree of satisfaction with the work carried out as a teacher during the lockdown period (Q8), in the case of women, 77% answered “Satisfied” or “Very satisfied”. In the case of men, this proportion was 70%. The percentage of people who scored “Little satisfied” or “Not at all satisfied” was 6% in the case of women and 9% in the case of men. See Figure 15.

#### 3.2.5. Research

**Figure 16:**
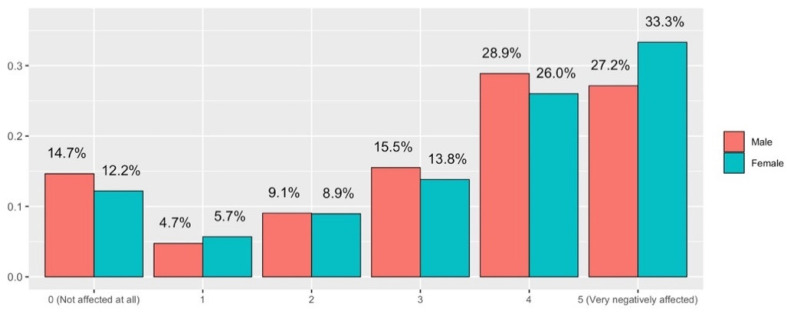
Answers to Q11 disaggregated by gender.

Regarding the impact on research due to online teaching (Q11), it can be seen (Figure 16) that the score of “4” or “5” adds up to exactly the same in both cases, but curiously in the case of men there are 14% of the responses stating that their research has been “Not affected at all” (answer with “0” value). In the case of women, there is no answer with a value of “0”. Perhaps the increase in dedication to virtual teaching has taken up time normally spent focusing on research. It is true that confinement prevents access to research laboratories, but we must assume that this impediment is common to both groups, regardless of men or women, and is therefore not the ultimate cause of this difference in responses. At first glance, women state that their research has been much more affected by the dedication required by non-contact teaching than in the case of men (the sum of “2”, “1” and “0” values exceeds the 25% for men).

### 3.3. Reconciliation and Research Related to Other Aspects

Since one of the objectives of this work is to analyze the impact of personal reconciliation and research during the pandemic period and above all its differences when it comes to gender, we analyze the relationship between the responses to these two aspects (Q3 and Q11) with the rest of the evaluated aspects, both in general and disaggregated by gender. Since variables Q1.a, Q1.b, Q1.c and Q4 are not ordinal variables, their answers have no comparative meaning; therefore, they have been eliminated from the analysis. Besides, as mentioned before, the question related to the Bachelor or Master Theses (Q7) had a diversity of answers with different casuistry, therefore it was also removed from this analysis.

The analysis is conducted through radar charts (or spider diagrams). These diagrams display multivariable data in a two-dimensional chart revealing the relationships, trade-offs and comparative measures. Each radar chart is a plot that consists of a sequence of equiangular spokes, with each spoke representing one of the variables. All spokes start at the same point representing a value equal to zero and each circumference indicates an increment of 1 in the measure in each variable. In each spoke a point is drawn which represents the mean of the answers, beside which the 95% confidence interval is represented by a straight line over the spoke. This interval gives us information about the variability of the response: the longer the line, the more variability the variable has.

**Figure 17:**
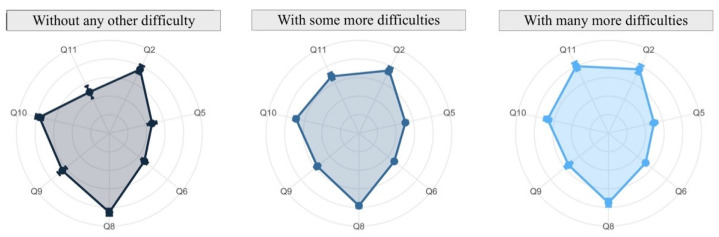
Reconciliation of virtual teaching activity with personal life compared to reconciliation in standard conditions (answers to Q3) related to the other aspects.

The relationship between the reconciliation of virtual teaching activity with personal life compared to reconciliation in standard conditions (answers to Q3) and the other aspects can be seen in Figure 17. It is clear that variables Q5, Q6, and Q11 are directly related to the degree of reconciliation. In other words, inquiries by email or by videoconference and their negative impact on research increases, making reconciliation more difficult. On the other hand, Q8, Q9 and Q10 have an inverse relationship, that is, despite having more difficulties in the reconciliation, the general satisfaction decreases.

**Figure 18:**
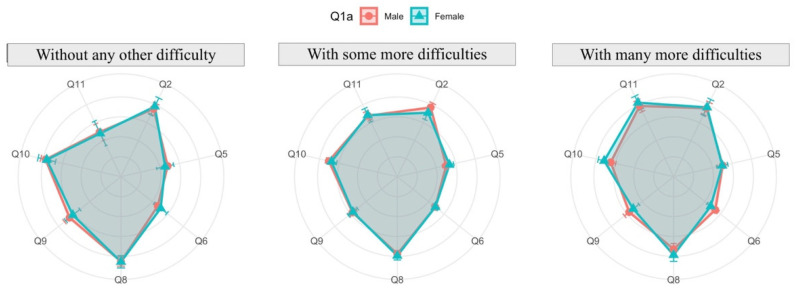
Reconciliation of virtual teaching activity with personal life compared to reconciliation in standard conditions (answers to Q3) related to other aspects and disaggregated by gender.

Disaggregating previous results by gender (Figure 18), the main difference is seen in those faculty who have revealed to have many more difficulties in the reconcialiation. Women considered themselves to have used less time in videconferences and covered less of the subject (Q6 and Q9), but they are more satisfied by their work and student learning (Q8 and Q10) and the negative impact on the research is higher than men.

**Figure 19:**
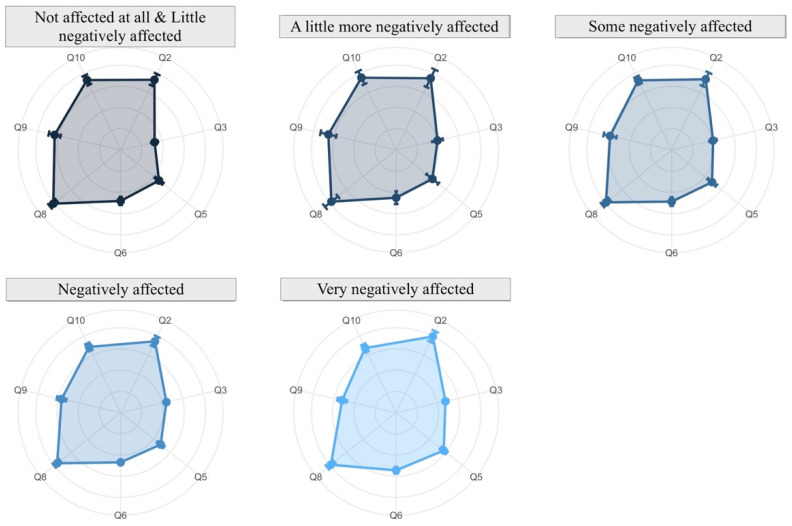
Affectation of research due to virtual teaching dedication (answers to Q11) related to other aspects.

On the other hand, the relationship between the answers related to affectation of research due to virtual teaching dedication and the rest of the aspects can be seen in Figure 19. A direct relationship of research with reconciliation (Q11) is confirmed; the more difficulties in reconciliation, the more negative the impact on research. An inverse relation is seen with Q6 and Q9; if the student consultations and percentage of subject covered decrease, the negative effect increases.

**Figure 20:**
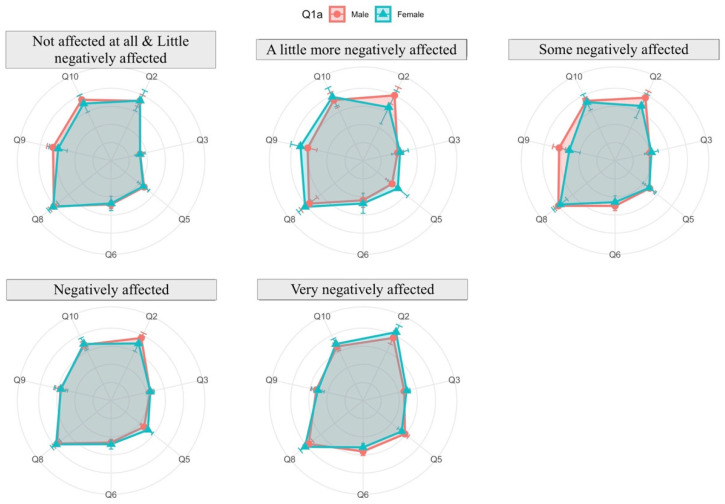
Effects on research due to virtual teaching dedication (answers to Q11) related to other aspects and disaggregated by gender.

Futhermore, disaggregating by gender, Figure 20 shows that from faculty staff who consider that their research has been very negatively affected, women state that they have dedicated more time to virtual teaching (Q2); despite this, they are more satisfied regarding their teaching work.

### 3.4. Statistical Analysis

The statistical significance of the data in this work has been analyzed, disaggregated by gender in the following questions: Q2 (How much more or less time do you spend on online teaching compared to face-to-face teaching in a normal situation?), Q3 (How do you reconcile teaching activity throughout the health crisis with personal life with respect to the normal situation?), Q8 (Are you satisfied with the work you have done as a teacher during this period of confinement?), Q10 (Are you satisfied with the learning achieved by your students?), and Q11 (Has your research been negatively affected by your dedication to virtual teaching?”).

Considering that the answers to the questions are not normally distributed, the two groups of samples (Female and Male) are considered as two independent groups to be compared. To do this comparison, an unpaired two-samples Wilcoxon test has been conducted whose results are shown in Table 4.

The Wilcoxon test showed that both samples, male and female, do not give statistical significance in questions Q10 and Q11, which seems logical since these are aspects in which gender does not seem to be a differentiating element. However, it is remarkable that in questions Q2, Q3 and Q8 the gender factor is very close to being a determining factor.

It is possible to carry out an analysis of the survey to see if there is a relationship between the different questions that have been asked. For this, the correlation coefficients between questions Q2, Q3, Q5, Q6, Q8, Q9, Q10 and Q11 have been determined. See Table 5.

From Table 5 it can be seen that the correlation coefficients obtained in all cases are far from the value of 1, so there is no relationship between the variables.

Considering variables are ordinal variables, the polychoric correlation is also calculated (see Table 6). Correlation values far from 1 can be observed.

### 3.5. Open Field Results

From the questionnaire, 110 of the respondents wrote a comment on the open field question at the end of the survey. The comments on the survey, by their very nature, were diverse and sometimes showed the most critical points for those who made them. They are statistically less significant than the rest of the survey responses but complement aspects that the other questions did not take into account. After applying constant comparison, six different codes emerged. Some responses refer to more than one code. The responses were classified into the following six categories: Teaching, Resources, Working conditions, Information, Research and Reconciliation. There were some comments that were not taken into account because they were not significant as there was only one. Of the groups analysed, the one with the most answers was Teaching with 32, and the one with the least was Reconciliation with 4. One of the sentences present in the comments has been included in the header of each topic and may be representative of what most sayed.

- Teaching: “The main problem is the practices that cannot be done in any way at a distance, in the subjects I teach” (32 comments)

There are issues, such as that internships and Bachelor/Master Theses with a laboratory load should be done in person, where the opinion was unanimous. There was also a consensus in advising face-to-face assessment, especially for first-year students. Regarding the non-face-to-face methodology, a good part of the comments indicated that it could be a good working tool that could complement or even replace face-to-face in some aspects, although there were some detractors who think that face-to-face teaching is irreplaceable.

- Resources: “Suppose the police had to patrol with private cars” (22 comments)

The authors collected 22 comments regarding the resources used to perform the work at home during the pandemic period. A large majority pointed out that they have had to work with personal equipment that they sometimes had to share with the rest of the family who also needed them, all because the resources provided by the university have been minimal or non-existent. There are people who have had to buy microphones, speakers, cameras, virtual whiteboards, and even cell phones, tablets and computers, all to perform the job as well as possible. Teachers also noted that working at home has led to the consumption of electricity, data and private communication services that should be recognised and compensated as is done in all companies where there is telework.

Using Google Meet^®^ platform has taken many hours of work and teachers would need to keep this tool for a while to recoup the time spent learning. A standard platform that works in a variety of environments would also need to be found. For example, there are people who have had to pay for Zoom^®^ licenses because Google Meet^®^ was not the best place to draw or because it did not work in the private environment due to operating system or browser issues.

Finally, the most consumed resource during this lockdown has been time; teachers have spent many hours in the preparation of non-contact material, in the monitoring of students and in the learning of tools and resources, all to reach the deadlines and achieve the highest quality teaching.

- Working conditions: “Counting even the time saved in travel, I work more hours than in person” (22 comments)

The university has not valued teaching for many years, and therefore, when this crisis arrived, it was not sufficiently prepared. The current situation has shown that the model of individual recognition with Teaching Activity Points is not fair as it does not consider the number of hours needed to prepare materials, to serve students, to coordinate, to update campuses, to train in non-contact tools, etc. In addition, the number of hours devoted to non-contact teaching depends on the number of students as the number of inquiries increases.

Continuous work for many hours and days has affected many people personally worsening the necessary rest, increasing stress and even worsening their own health (back and joint pain, vision problems, dizziness, leg pain, etc.).

There were many comments from Adjunct Professors who complain that they have had to work much harder than they have hired and acknowledging that they should have spent even more hours to deliver quality teaching, but their external professional work prevented it, all this for a rather meager salary. The only thing that has partially offset them is the reduction in transportation costs and the number of hours spent on travel.

- Information: “ICT staff have done everything they can” (seven comments)

There were few comments regarding Information and Communications Technology (ICT) support and the information received. Those present commented that sometimes the information provided, both in terms of emails and possible tools to be used, has been of little use due to the large amount of information given without prior screening. The work done by ICT staff is acknowledged, but at the same time, better online ICT support is requested, such as a telephone line to resolve doubts.

- Research: “Impossible to reconcile research and non-face-to-face teaching in the current conditions” (seven comments)

Many comments denounce the impossibility of devoting time to research due to the large number of hours devoted to non-contact teaching and the impossibility of accessing research laboratories. The need to reconcile the avalanche of work with personal life has also made it difficult to dedicate oneself to research. Others comment that they have been able to do something as long as it does not require a presence in the laboratory (articles, proofreading, preparation of proposals, etc.).

- Reconciliation: “The main problem is personal reconciliation with two small children at home” (four comments)

The comments came from those who have young children at home who, during the confinement, have logically required constant attention, a rather complicated job to combine with the preparation of teaching materials, attention to a multitude of emails, long and continuous videoconferences, etc.

## 4. Discussion

The results show that the Universitat Politècnica de Catalunya faculty has notably increased their academic activity due to the number of hours devoted to virtual teaching compared to their usual teaching time, as Twigg [73] highlights. More than half (57.2%) of the academic staff said their research had been negatively or very negatively affected. It is important to note that women stated that their research had been much more affected by the dedication that virtual teaching requires than in the case of men, as Krukowsli et al. [62] remark. 74% of part-time teaching staff (with working conditions that depend on other external activities), who represent a significant part of the teaching staff, responded that the increase in their teaching dedication had been equal to or greater than 50%. A general malaise was seen among the teaching staff due to the fact that the institution did not worry or ask if they had the minimum equipment and infrastructure to be able to carry out their work virtually; this fact was mentioned in other studies such as [74].

Sharma et al. [75] state that the teaching and research staff have faced many challenges during the pandemic period. One of the main aspects to consider, as Marek et al. [76] highlight, is the high workload that faculty staff experienced when converting face-to-face classes to distance learning. The environment in which online teaching takes place is different from that in traditional learning. To carry out online teaching, teachers should not only focus on the technological specifications of the tools, but they also need to learn how to use new software, turn the course into electronic version and adjust the pace of the class, as Van der Rijst et al. [77] report. The survey highlights that teachers have spent many hours in the preparation of material, in the monitoring of students and in the learning of tools and software, all to achieve the highest quality teaching. Moreover, faculty needed to adapt the evaluation methods [78]. Conducting assessments remotely during COVID-19 has posed extraordinary challenges for higher education teachers owing to lack of preparation superimposed with the inherent problems of remote assessment.

A general malaise was seen among the teaching staff due to the fact that the institution did not worry or ask if they had the minimum equipment and infrastructure to be able to carry out its work virtually; a large majority pointed out that they have had to work with personal equipment to do the job as well as possible. This crisis has shown that all teachers should be provided with the basic material to teach and highlighted that educational institutions need to provide resources when returning to traditional education or in response to a future crisis. Otherwise, this could be translated in a decrease of the level of satisfaction from academic staff work, together with the lack of contact with students and co-workers, as Szromek et al. [79] report. Training for faculty staff is also important, and depends on education policymakers that pay attention to experienced instructors, being able to prepare teachers’ competencies to teach in a virtual learning environment, to renew assessment strategies and teaching methodologies to improve emergency preparedness [5,41,80].

More than half (57.2%) of the academic staff said their research had been negatively or very negatively affected. In addition to the increase in academic activity due to the number of hours dedicated to virtual teaching, the negative impact on research may be related to the temporary closure of scientific laboratories, as Back et al. [81] state. Another issue that faculty remarked was that internships or Bachelor/Master Theses with a laboratory load were difficult to conduct since they should be done in person, and the same was valid for the practices of some subjects that cannot be done remotely in any way. The research workforce is contending with the need to develop new online learning resources for teaching and increased domestic responsibilities associated with closures of schools and child-care facilities [7]. The main problem was personal reconciliation for those with children at home, who, during the confinement, have logically required constant attention, which has especially affected their teaching and the research work. As pointed in [82], the biggest impact on faculty staff was for the most precarious professional stages or those with care responsibilities. Apart from that, the pandemic has provided an opportunity for faculty staff to consider how they optimize academic productivity and how their work lives will be structured in the future [83,84].

Regarding results disaggregated by gender, from the question of the survey about job categories, the vertical segregation by gender in academia, that is, the under-representation of women in the highest job categories, is evident; as [57,69,85] state, women seem to be stuck in the Associate Professor (Statutory and Contracted) categories (see Figure 11). Virtual activity has been different for women and men; as the survey shows, women were more likely to state that they had more difficulties in reconciling teaching activity with personal life throughout the health crisis compared to men’s responses, as stated in [46,56,57]. They referred to responsibilities of caring for children and/or dependent people. From the survey it can be seen that women have spent more time in virtual teaching compared to face-to-face teaching than men. This corresponds with the satisfaction with the work done as a teacher during lockdown, as women answered that they were more satisfied with their work than men did. However, it is important to highlight that women stated that their research had been much more negatively affected than in the case of men, as other studies point that during the pandemic, female academic productivity has been lower than male academic productivity, as Krukowski et al. [62] state. Taking into account the above comments, this can be due to women’s higher dedication in virtual teaching and that women devote significantly more time to household work than do men. It is obvious that a decrease in female research will lead to an increase in the gender gap in academia, as Iwasaki et al. [86] show. All these disparities show that confinements are strongly impacting women that are more vulnerable to the effects of a crisis (see [87]). These data should be considered by higher education institutions when making decisions about hiring, as well as promotion and tenure.

## 5. Conclusions

The teaching and research staff has significantly increased its academic activity due especially to the number of hours devoted to virtual teaching compared to its teaching dedication in situation of normalcy, which has led to a very high workload and stress situation. These circumstances and the fact that it was not possible to access the research laboratories for weeks has had a negative impact on academic productivity. This negative impact on research has been higher in women, who already face disparities regarding promotion and, during lockdown, stated more difficulties with personal life reconciliation.

Of particular concern is the increase in time dedicated by part-time teaching staff (Adjunct Professors), who cover an important part of teaching with part-time working conditions that depend on other external work activities. From the respondents, 74% of Adjunct Professors or part-time teaching staff (with working conditions that depend on other external activities), who represent a significant part of the teaching staff, stated that the increase in their teaching dedication had been equal to or greater than 50%. All this was done for a rather meager salary of teaching staff. This makes it clear that it is necessary to rethink the hiring model at higher education institutions.

Weaknesses in online teaching infrastructures need to be further explored and deficiencies and inexperienced knowledge of teachers should be better understood, including uneven learning outcomes caused by varied experience. There is widespread unease with the fact that the institution did not worry or ask the teaching and research staff if they had the minimum of equipment and the minimum network infrastructure to be able to carry out work in person. The teaching and research staff has carried out their activity, pouring in personal resources and with a dedication far beyond the normal working day.

The workload and the stress endured has been much greater than in the face-to-face classes, but what was necessary has been done to serve the students through quick adaptability and good planning. In general, the degree of satisfaction with the work done is high, as the goal of getting the course going and making the students feel well taken care of has been achieved. There are some comments that question the evaluation mechanisms used and the impossibility of controlling fraudulent actions.

Uncertainties will continue in the future. Many subjects will have an important non-face-to-face part or will be done completely non-face-to-face. There is also the possibility of having to face new lockdowns. Given the experience of recent months, we believe it is necessary to undertake the following actions:

- Ask for recognition of the non-contact work of the teaching and research staff. It is necessary to recognise the hours that the teaching and research staff will dedicate to this task. It is also necessary to increase the teaching capacity of the university to cope with the increase in teaching work that involves non-contact teaching. This fact must be reflected in the university’s hiring model.
- A budgetary allocation is needed in order to be able to sufficiently equip the teaching and research staff who work from home. In addition, since faculty staff will have to combine face-to-face and non-face-to-face teaching, it is also necessary to provide the teaching and research staff with the appropriate material so that they can do non-face-to-face teaching in the workplace. It is absolutely essential to ask the teaching and research staff what they need, to offer advice on the material that best suits their needs and to finance the purchase of this material.
- All teaching and research staff evaluation and accreditation processes related to staff promotion, both internal and external to the university, which take into account the research developed, in a time equivalent to that of the lockdown period should not be considered for evaluation, unless the person concerned states otherwise.

## Figures and Tables

**Table 1:** Job categories of the teaching and research staff of the Universitat Politècnica de Catalunya.

Teaching and Research Staff		
**Statutory Teaching and Research Staff**	Full Professors Associate Professors	
	University School Full Professors	
	University School Associate Professors	
**Contractual Teaching and Research Staff**	Permanent	Contracted Full Professors
		Contracted Associate Professors
		Collaborating Professors
	Temporary	Assistant Professors
		Temporary Collaborating Professors
		Assistant Staff
		Adjunct Professors
**Research Staff**		
**Other**		

**Table 2:** Most important design aspects of the survey.

Survey	Description
Type of survey	Transversal
Population	Teaching and research staff
Confidence interval	95%
Sampling error	0.02%
Survey period	June 2020
Sample	2885 faculty (355 answers, 12.3%). Voluntary non-probabilistic
Process	Anonymous online
Data collection instruments	Google Forms^®^
Data analysis instruments	IBM SPSS v19 Solutions for Education^®^

**Table 3:** Survey questions.

Survey Questions (Question Number)	Answers
Gender (Q1.a)	“Female”
	“Male”
	“Other”
Age (Q1.b)	“Less than 30”
	“30–39”
	“40–49”
	“50–59”
	“More than 60”
Job category (Q1.c)	“Full professor”
	“Associate professor”
	“University School Associate Profesor”
	“Adjunct Professor”
	“Contracted Full Professor”
	“Contracted Associate Professor”
	“Assistant Professor”
	“Collaborating Professor”
	“Other”
How much more or less time do you spend on virtual teaching compared to face-to-face teaching in a normal situation? (Q2)	“The same”
	“25% more”
	“50% more”
	“75% more”
	“100% or even more”
	“25% less”
	“50% less”
	“75% or even less”
How do you reconcile teaching activity throughout the health crisis with personal life with respect to the normal situation? (Q3)	“With many more difficulties”
	“With some more difficulties”
	“Wthout any other difficulty”
Of the following equipment or materials, indicate those that you consider NOT suitable to carry out your teaching activity from your home and that should therefore be improved. (Q4)	“Computer”
	“Software”
	“WebCam”
	“Table and chair”
	“Microphone or speaker”
	“Good network connection”
	“Room or office”
	“Other”
In this period of virtual teaching, the increase in student inquiries by email has been: (Q5)	“They have not increased”
	“The increase was less than 50%”
	“The increase was between 50 and 100%”
	“The increase was over 100%”
The weekly hours (H) devoted to student inquiries by video conference have been: (Q6)	“Zero hours”
	“From 0 to 3 h per week”
	“From 3 to 6 h per week”
	“More than 6 h per week”
If you are a Bachelor/Master Thesis tutor, have you had to increase the frequency of inquiries to ensure the development of the Bachelor/Master Thesis? consultations for Bachelor/Master Thesis (Q7)	“It has not increased”
	“It has doubled”
	“It has tripled”
Are you satisfied with the work you have done as a teacher during this period of confinement? (Q8)	“Very satisfied”
	“Satisfied”
	“Neither satisfied nor dissatisfied”
	“Little satisfied”
	“Not at all satisfied”
With respect to previous academic years, what percentage of the subject did you manage to teach with the same depth? (Q9)	“Less than 80%”
	“80%”
	“90%”
	“100%”
	“110%”
	“More than 110%”
Are you satisfied with the learning achieved by your students? (Q10)	“Very satisfied”
	“Satisfied”
	“Neither satisfied nor dissatisfied”
	“Little satisfied”
	“Not at all satisfied”
Has your research been negatively affected by your dedication to virtual teaching? (Q11)	“Very negatively affected”
	“Negatively affected”
	“Some negatively affected”
	“A little more negatively affected”
	“Little negatively affected”
	“Not affected at all”
Add any relevant comments or aspects if you consider it necessary.	Open field answer

**Table 4:** Mean values $\bar{x}$ and *p* values for questions Q2, Q3, Q8, Q10 and Q11.

Question	$\bar{x}$	*p*
Q2	$\bar{x}\;female=3.565573$ $\bar{x}\;male\;=3.415584$	0.0943
Q3	$\bar{x}\;female=1.163934$ $\bar{x}\;male\;=1.077922$	0.0609
Q8	$\bar{x}\;female=4.016393$ $\bar{x}\;male\;=3.887745$	0.0868
Q10	$\bar{x}\;female=3.540983$ $\bar{x}\;male\;=3.519480$	0.3677
Q11	$\bar{x}\;female=3.352459$ $\bar{x}\;male=3.220779$	0.179

**Table 5:** Correlation coefficients between questions Q2, Q3, Q5, Q6, Q8, Q9, Q10 and Q11.

	Q2	Q3	Q5	Q6	Q8	Q9	Q10	Q11
**Q2**		0.24140809	0.23527968	0.14617751	0.03641864	−0.06463380	−0.07969498	0.28617004
**Q3**			0.04467434	0.06879415	−0.18202458	−0.14643627	−0.17058812	0.35739666
**Q5**				0.31236712	0.05423561	−0.04781707	0.01960635	0.17592060
**Q6**					−0.01016529	−0.00876383	0.01471297	0.12933004
**Q8**						0.44209437	0.57130368	−0.06184509
**Q9**							0.44512755	−0.20297173
**Q10**								−0.13856441
**Q11**								

**Table 6:** Polychoric correlation coefficients between questions Q2, Q3, Q5, Q6, Q8, Q9, Q10 and Q11.

	Q2	Q3	Q5	Q6	Q8	Q9	Q10	Q11
**Q2**		0.07384518	0.05475970	0.076799156	0.149907813	−0.05360012	0.07673716	0.17881187
**Q3**			0.04656107	0.092313773	−0.202108152	−0.15779416	−0.19617641	0.43514855
**Q5**				0.351461975	0.066220039	−0.05419668	0.02258320	0.24136142
**Q6**					0.009511047	−0.02077117	0.03493697	0.18517624
**Q8**						0.45289464	0.60588946	−0.02420343
**Q9**							0.47470316	−0.21592029
**Q10**								−0.14206301
**Q11**								

## Data Availability

Data and material available on request from the authors. The data and materials that support the findings of this study are available from the corresponding author upon reasonable request.
